# Production of oleanane-type sapogenin in transgenic rice via expression of *β*-amyrin synthase gene from *Panax japonicus* C. A. Mey

**DOI:** 10.1186/s12896-015-0166-4

**Published:** 2015-06-02

**Authors:** Zhiwei Huang, Juncheng Lin, Zuxin Cheng, Ming Xu, Mingshu Guo, Xinying Huang, Zhijian Yang, Jingui Zheng

**Affiliations:** College of Food Science, Fujian Agriculture and Forestry University, 15 Shangxiadian Road, CangShan District, Fuzhou, 350002 Fujian China; Agricultural Product Quality Institute, Fujian Agriculture and Forestry University, 15 Shangxiadian Road, CangShan District, Fuzhou, 350002 Fujian China

**Keywords:** Ginsenosides, Sapogenin, *β*-amyrin synthase gene, Rice, Genetic transformation

## Abstract

**Background:**

*Panax japonicus* C. A. Mey. is a rare traditional Chinese herbal medicine that uses ginsenosides as its main active ingredient. Rice does not produce ginsenosides because it lacks a key rate-limiting enzyme (*β*-amyrin synthase, *β*AS); however, it produces a secondary metabolite, 2,3-oxidosqualene, which is a precursor for ginsenoside biosynthesis.

**Results:**

In the present study, the *P. japonicus βAS* gene was transformed into the rice cultivar ‘Taijing 9’ using an *Agrobacterium*-mediated approach, resulting in 68 rice transgenic plants of the T_0_ generation. Transfer-DNA (T-DNA) insertion sites in homozygous lines of the T_2_ generation were determined by using high-efficiency thermal asymmetric interlaced PCR (hiTAIL-PCR) and were found to vary among the tested lines. Approximately 1–2 copies of the *βAS* gene were detected in transgenic rice plants. Real-time PCR and Western blotting analyses showed that the transformed *βAS* gene could be overexpressed and *β*-amyrin synthase could be expressed in rice. HPLC analysis showed that the concentration of oleanane-type sapogenin oleanolic acid in transgenic rice was 8.3–11.5 mg/100 g dw.

**Conclusions:**

The current study is the first report on the transformation of *P. japonicus βAS* gene into rice. We have successfully produced a new rice germplasm, “ginseng rice”, which produces oleanane-type sapogenin.

## Background

Rice is one of the world’s major food crops. It is the staple food for nearly half of the world’s population [1]. Traditional breeding techniques, especially hybrid rice technology, have contributed to the improvement of rice yield and quality. However, there are compounds that rice cannot synthesize or can only produce at very low levels, and traditional breeding techniques are apparently incapable of resolving these problems. Transgenic technology provides an efficient means of improving rice quality at the genetic level [2–4] based on the principle that creating a new rice germplasm that is capable of producing exogenous active ingredients through genetic engineering could improve the nutritional quality of rice. For example, Ye *et al.* [5] and Paine *et al.* [6] produced the first and second generations of “golden rice”, respectively, mainly by expressing exogenous key genes in the β-carotene biosynthesis pathway in the endosperm. The reported concentration of β-carotene was 1.6 μg/g and 37 μg/g in these two transgenic rice strains, respectively. Lee *et al.* [7] transformed the sesame 2S albumin gene into rice and produced “sesame nutrition rice”, and the methionine content of the seeds reached 0.40% in the T_2_ generation rice lines, which represented a 38% increase relative to that of the control rice lines.

**Fig. 1:**
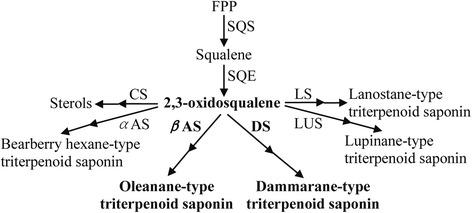
Biosynthetic pathway of triterpenoid saponin and sterols in plants [10–12]. Intermediates: FPP, farnesyl pyrophosphate; SQS, squalene synthase; SQE, squalene epoxidase; CS, cycloartenol synthase; LS, lanosterol synthase; *α*AS, *α*-amyrin synthase; LUS, lupeol synthase; DS, dammarenediol-II synthase; *β*AS, *β*-amyrin synthase

*P. japonicus* is a rare traditional Chinese herbal medicine that uses ginseng saponin as its main active ingredient. Its health-protective effects include improving immunity, preventing tumors, and facilitating adaptation [8, 9]. The total saponin content in the roots of *P. japonicus* can reach 15%, which is 2- to 7-fold higher than that of *P. ginseng* and 3-fold higher than that of *P. quinquefolius* [8, 9]. Ginsenosides are triterpenoid saponins of plant secondary metabolites, *i.e.* the product of triterpenoid saponins biosynthesis branch in the isoprenoid pathway (Fig. 1) [10–12].

Triterpenoid saponins are formed by different cyclization reactions of squalene. The synthesis of squalene is the branch point of the central isoprenoid pathway. It occurs at the triterpenoid saponin biosynthetic branch [11, 13, 14]. 2, 3-oxidosqualene cyclases (OSCs), the rate-limiting enzymes in triterpenoid saponins and sterols biosynthesis branches, catalyze 2,3-oxidosqualene, which results in the formation of the triterpenoid skeleton, cycloartenol, and other compounds. With further modifications, these compounds can form a variety of triterpenoid saponins, phytosterols, and other macromolecules.

The type and catalytic function of OSCs differ between ginseng plants and rice. The OSCs of ginseng plants are mainly *β*-amyrin synthase (*β*AS) and dammarenediol-II synthase (DS). These catalyze 2,3-oxidosqualene to produce oleanane-type and dammarane-type substances, namely, *β*-amyrin and dammarenediol-II, respectively. Rice OSC is a cycloartenol synthase (CS) that catalyzes 2,3-oxidosqualene to produce cycloartenol [6, 11, 14]. Zhao *et al.* [15] studied the expression of ginseng *βAS* and the regulation of ginsenosides biosynthesis by using antisense RNA technology. When antisense *βAS* was introduced into the hairy roots of ginseng, the transcription levels of the *βAS* gene significantly decreased compared to those of non-transgenic controls. In addition, *β*AS activity also decreased, and the concentration of oleanane-type ginsenosides Ro decreased by as much as 40%. DS activity increased in these *βAS* antisense lines, and the concentration of dammarane-type ginsenosides increased by up to 30%. These results proved that regulation of ginsenosides synthesis could be achieved by altering the gene expression pattern of *βAS* by using genetic engineering techniques.

The *βAS* gene has been cloned from *Panax ginseng* (GenBank Acc. Nos.: AB014057.1, AB009030.1) [10], *Aralia elata* (GenBank Acc. No.: HM219225.1), *Betula platyphylla* (GenBank Acc. No.: AB055512.1), *Malus domestica* (GenBank Acc. No.: FJ032007.1), *Pisum sativum* (GenBank Acc. No.: AB034802.1) [10], and *Centella asiatica* (GenBank Acc. No.: AY520818.1). The *βAS* gene has not been cloned from rice. However, rice OSC is a CS, which catalyzes 2,3-oxidosqualene to produce the sterol substrate, cycloartenol [10, 16–18]. This indicates that although 2,3-oxidosqualene, the precursor of rice sterols synthesis, is also the precursor for ginsenosides synthesis [11, 13], rice cannot synthesize ginsenosides because rice lacks *β*AS and DS, which are the rate-limiting enzymes in the synthesis of ginsenosides.

In summary, genetic engineering of the secondary metabolic pathways allows rice to synthesize saponin. However, no report on the use of genetic engineering on rice to synthesize saponin currently exists. Previously, our research group isolated the full-length cDNA of *βAS* (GenBank Acc. No.: KP658156) from the roots of *P. japonicus*, cloned this into the pMD® 18-T Simple Vector (Takara, D103A), and named the construct as pMD-AS.

To create a new “ginseng rice” germplasm and to provide germplasm resources for further improvement of rice quality, the present study transformed the *P. japonicus βAS* gene into rice to synthesize oleanane-type sapogenin.

## Methods

### Experimental materials

Rice cultivar ‘Taijing 9’, *Escherichia coli* (*E. coli*) strain DH5α, *Agrobacterium tumefaciens* (*A. tumefaciens*) strain LBA4404, plasmids pMD-AS, pMD-Gt1-AmA1, pBlue-Ubi, and pCD-AMA1-hpt were maintained by the Agricultural Product Quality Institute of Fujian Agriculture and Forestry University.

### Construction of a binary plant expression vector carrying the *βAS* gene

**Fig. 2:**
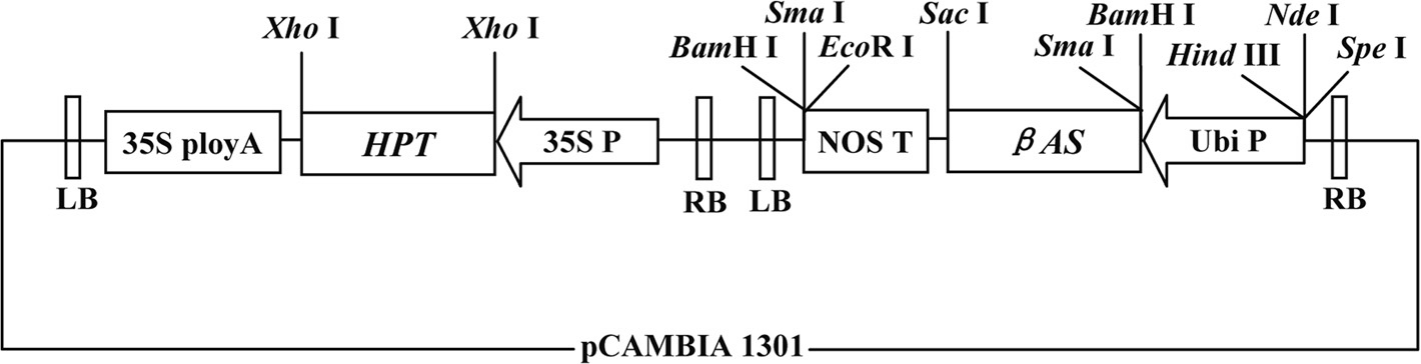
T-DNA region of the plant expression vector pCD-AS-hpt. Intermediates: 35S polyA, terminator of *CaMV* 35S gene; *HPT*, hygromycin phosphotransferase gene; 35S P, 35S promoter; NOS T, terminator of nopaline synthase gene; Ubi P, Ubiquitin promoter; LB, left border; RB, right border

The construction of a binary plant expression vector harboring the *βAS* gene was as described elsewhere [19]. Briefly, double digestion of plasmids pMD-AS by *Sma*I and *Sac*I was conducted to isolate a DNA fragment that included the *βAS* gene. The fragment was then recovered and ligated to *Sma*I and *Sac*I double-digested pMD-Gt1-AmA1 vector to form pMD-Gt1-AS. The pBlue-Ubi plasmid was double-digested with *Hind*III and *Bam*HI to release the Ubi promoter fragment. This fragment was then recovered and ligated to *Hind*III and *Bam*HI double-digested pMD-Gt1-AS vector to form plasmid pMD-Ubi-AS. The pMD-Ubi-AS plasmid was double-digested with *Hind*III and *Sac*I to release the Ubi-*βAS* fragment. This fragment was then recovered and ligated to *Hind*III and *Sac*I double-digested plasmid pCD-AMA1-hpt to form binary plant expression vector, pCD-AS-hpt. The structure of pCD-AS-hpt is shown in Fig. 2. The recombinant vector pCD-AS-hpt was confirmed using *Hind*III/*Sac*I and *Hind*III digestion, and the plasmid was sequenced to determine that no mutations in the target gene were present.

### Transformation of binary *βAS* plant expression vector into rice

Inflorescences of rice ‘Taijing 9’ at 10–15 days after pollination were collected based on the method of Chen *et al.* [20] to induce embryogenic callus and subculture callus once every 15 days with fresh medium.

Using the freeze-thaw method [21], the binary expression vector pCD-AS-hpt was transformed into *A. tumefaciens* LBA4404, which was used to transform embryogenic calli. Co-culture of callus with *Agrobacterium*, screening of resistant calli and induction of differentiation of cultured calli, rooting of resistant seedlings, and other tests were conducted as described by Burkhardt *et al.* [22]. The medium used for Agrobacterium infection was AAM medium (pH 5.2). NB medium (pH 5.8) was used for genetic transformation of rice. To screen for resistant calli, a culture medium supplemented with 50 mg/L hygromycin B (Hyg) was used [22].

### Identification of transgenic rice by PCR

Genomic DNA was extracted from leaves of resistant seedlings of transgenic rice. *Premix Taq*® Version 2.0 (Takara, D334A) was used to detect the *βAS* and hygromycin resistance (*HPT*) genes in the transgenic rice plants. The primers used in the present study were as follows: ASF5 (5′-ATGCTTGCTTGTTGGGTTGAGG-3′), ASR2 (5′-GCCTGAATTGCTGATGAAGTGC-3′), Hpt-1 (5′-TACACAGCCATCGGTCCAGA-3′), and Hpt-2 (5′-TAGGAGGGCGTGGATATGT C-3′). The PCR reaction system included a 25-μL solution containing 12.5 μL *Premix Taq*® Version 2.0 (+dye), 0.5 μL of each of the forward and reverse primers (20 μM), 2.0 μL of DNA template, and 9.5 μL of ddH_2_O. The PCR reaction conditions were as follows: 94 °C for 5 min; 94 °C for 30 s, 58 °C for 30 s, and 72 °C for 60 s/kb for 35 cycles; and 72°C for 10 min.

### Analysis of T-DNA insertion sites by hiTAIL-PCR

High-efficiency thermal asymmetric interlaced PCR (hiTAIL-PCR) is a convenient way of locating T-DNA insertion sites [23]. Leaf genomic DNA was extracted from rice homozygous lines of the T_2_ generation. Based on the methods described by Liu *et al.* [22], hiTAIL-PCR was conducted by using *Premix Ex Taq*® Version 2.0 (Takara, D335A) to amplify genomic sequences flanking the T-DNA insertion sites in transgenic rice. The clear bands that had good specificity and were > 200 bp in size for the third PCR product were recovered for sequencing. PCR verification of the T-DNA flanking sequences was conducted by using 2× *Eco* Taq PCR SuperMix (TransGen Biotech, AS151). The forward primer was RB-3b (5′-GATCGCCCTTCCC AACAGTTGC-3′), and the reverse primers were A30R (5′-CAACACCCACATCGCCATCTG C-3′), A37R (5′-GCCGAGACCATCGATTCCAATG-3′), A43R1 (5′-GGCGTATGTCTCATT GGAGGACTGC-3′), and A43R2 (5′-CCAAGTACAGCCAACACTGCTGGTC-3′). The PCR reaction system included a 50-μL solution containing 25.0 μL of 2× *Eco* Taq PCR SuperMix (+dye), 1.0 μL of each of the forward and reverse primers (20 μM), 2.0 μL of the DNA template, and 21.0 μL of ddH_2_O. PCR reaction conditions were 94 °C for 5 min; 94 °C for 30 s, 58 °C for 30 s, and 72 °C for 60 s/kb for 35 cycles; and 72 °C for 10 min. The PCR products were recovered for sequencing. The resulting DNA sequences were then aligned to the rice ‘Nipponbare’ genome sequence (http://rice.plantbiology.msu.edu) by using the BLASTN program to determine the insertion sites and copy number of T-DNA in transgenic rice.

### Determination of *βAS* expression levels using real-time PCR

Total RNA was isolated from the leaves of homozygous rice lines of the T_2_ generation by using TRIzol™ (Invitrogen, 15596026). Residual DNA was removed from the RNA samples by DNase I treatment (Takara, D2215). The reaction of DNase I treatment was as follows: 20–50 μg of total RNA, 5 μL of 10 × DNase I Buffer, 2 μL (10 units) of DNase I (RNase-free), 20 units of an RNase inhibitor, and DEPC-treated water to make up a total volume of 50 μL. DNase I treatment was conducted at 37 °C for 30 min, and the subsequent steps were conducted as recommended by the manufacturer. A PrimeScript® RT Reagent Kit (Takara, DRR037A) was used to reverse-transcribe total RNA to synthesize the first-strand cDNA. The conditions for reverse transcription reaction were as follows: 37 °C for 15 min and 85 °C for 5 s. SYBR® *Premix Ex Taq*™ II (Takara, RR041A) and the ABI 7500 real-time PCR System were used for *βAS* real-time PCR. Primers were qASF (5′-TGCCAGAGCAAGAAAATGGA-3′), qASR (5′-CATAGGAAGGAAAGGAGGAAGGA-3′). *ACTIN* served as the reference gene, and the primers were qACTF (5′-CATCTTGGCATCTCTCAGCAC-3′) and qACTR (5′-AACTTTGTCCACGCTAATGAA-3′). The PCR reaction system was a 25-μL solution containing 12.5 μL of 2× SYBR® *Premix Ex Taq*™ II, 0.5 μL of each of the forward and reverse primers (20 μM), 0.5 μL of 50× ROX Reference Dye II, 5.0 μL of first strand cDNA (diluted 5 times), and 6.0 μL of ddH_2_O. The PCR reaction conditions were as follows: 95 °C for 30 s; and 95 °C for 5 s and 60 °C for 34 s for 40 cycles. The relative expression of the *βAS* gene was RQ = 2^-ΔΔCt^. Each sample was amplified in triplicate.

### Western blot analysis of the *β*AS protein

Total protein was extracted from seeds of the T_2_ generation homozygous rice lines using RIPA lysis buffer (Beijing DingGuo Changsheng Biotechnology Company, Ltd., China), and total protein concentration was determined by using a total protein assay kit (Biuret Method, Shanghai Rongsheng Biotechnology Company, China). A 20–40 μg aliquot of the total protein solution was subjected to SDS-PAGE analysis by using a Mini-PRO TEAN® 3 Cell (BioRad). The Proteins were then transferred to a PVDF membrane by using a Trans-Blot SD semi-dry electrophoretic transfer cell (BioRad) and transmembrane buffer (48 mmol/L Tris base, 39 mmol/L glycine, 0.037% sodium dodecyl sulfate (SDS), and 20% methanol). The PVDF membrane was transferred to the TBST buffer (10 mmol/L Tris–HCl, 100 mmol/L NaCl, and 0.2% Tween-20, pH7.4) containing 5% non-fat milk and incubated at 4 °C overnight. After incubation, the PVDF membrane was reacted with the primary antibody (antiserum of the rabbit immunized with the *βAS* gene product expressed in *E. coli*) diluted in TBST buffer containing 5% non-fat milk (v/v 1:500) and incubated at 4 °C overnight. The PVDF membrane was then washed with TBST buffer. The secondary antibody (HRP-labeled goat anti-rabbit IgG, Beijing DingGuo Changsheng Biotechnology Company, Ltd., China) was diluted with TBST buffer (v/v 1:5,000), and the PVDF membrane was transferred to the above solution and incubated at room temperature for 1 h. The PVDF membrane was then washed with TBST buffer. The hybridization signal was developed by using the SuperSensitive ECL-solution (Pierce) and exposed for 1 min by using an X-ray film (Kodak) in a dark room [19]. Rabbit polyclonal antibody against *β*AS (primary antibody) was prepared by GL Biochem Co., Ltd. (Shanghai, China).

### Analysis of sapogenin content in transgenic rice by HPLC

The rice grains from the T_2_ generation homozygous rice lines were ground to powder and passed through an 80-mesh screen. Approximately 0.2 g of each sample was then transferred into a round flask to which 4 mL of methanol was added. The round flask was connected to a Dimroth’s condensing tube and reflux extraction was conducted in an 80 °C water bath for 12 h. Methanol was added to the extraction solution to a final volume of 5 mL. The solution was filtered through a 0.45-μm organic filter membrane and then subjected to HPLC analysis [24, 25].

The LC-20A high-performance liquid chromatography, SPD-M20A UV–vis detector and the software LC solution 1.24 SP1 (Shimadzu, Japan) were used to determine the concentration of sapogenin using oleanolic acid (Ole) as the standard. The column was a Hypersil ODS2 C18 column (250 nm × 4.6 nm, 5 μm; Dalian Elite Company, China), the chromatographic conditions were as follows: mobile phase was methanol - 0.05 mol/L NaH_2_PO_4_ (85:15), flow rate was 1.0 ml/min, column temperature was 30 °C, and the detection wavelength was 210 nm [24–26]. Standard oleanolic acid (Batch No.: 110709–200505) was purchased from the Chinese Academy of Food and Drug Testing.

## Results and discussion

### Generation of transgenic rice

The digestion products of the binary plant expression vector pCD-AS-hpt were of the expected sizes. Sequencing of pCD-AS-hpt showed no mutations in the *βAS* gene. These findings were indicative of the successful construction of the binary plant expression vector pCD-AS-hpt.

**Fig. 3:**
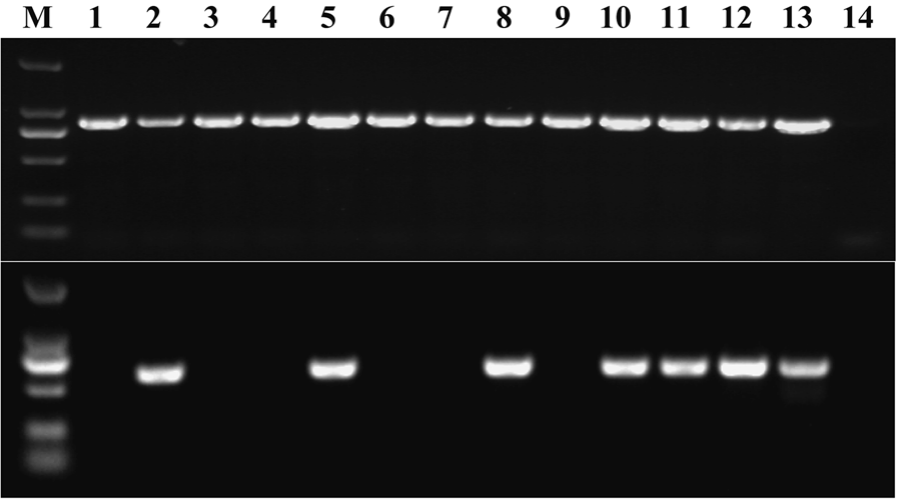
PCR analysis of *HPT* (845 bp) and *βAS* (584 bp) in transgenic rice plants. M, DNA Marker DL 2000. (1–12) Transgenic rice plants. (13), positive control (vector carrying the *βAS* gene). (14) Negative control (rice cultivar ‘Taijing 9’)

The binary vector was transferred to rice ‘Taijing 9’, and 302 anti-Hyg rice plants of the T_0_ generation were generated. The PCR results of *HPT* and *βAS* (Fig. 3) indicated that all resistant seedlings contained the *HPT* gene, whereas 68 plants contained *βAS* (positive rate: 22.5%). No significant differences in agronomic traits or appearance between the offspring of transgenic rice and the receiver ‘Taijing 9’ were observed.

### Confirmation of T-DNA insertion sites by hiTAIL-PCR

**Fig. 4:**
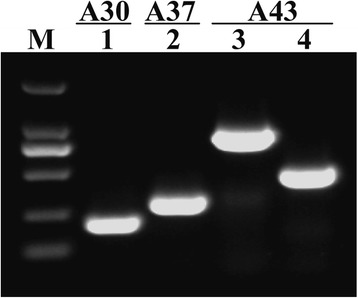
PCR analysis of T-DNA flanking sequences from transgenic rice plants. M, DNA Marker DL 2000. (1–4), PCR products of the T-DNA flanking sequences

hiTAIL-PCR analysis showed clear bands with good specificity in the third round of PCR products from three lines of the T_2_ generation homozygous rice lines, whereas that of the other lines were vague or smaller than 200 bp. Sequencing (GenBank Acc. Nos.: KP687751, KP687752, KP687753, and KP687754), PCR verification (Fig. 4), and alignment with the rice ‘Nipponbare’ genome sequence showed that the transgenic rice lines A30 and A37 both had one copy of the *βAS* gene-harboring T-DNA, whereas line A43 had two copies of the transgene. The insertion sites in these three lines were located at 9,533,306 bp in chromosome 5, 5,429,409 bp in chromosome 12, and 32,736,409 bp and 32,740,184 bp in chromosome 2, respectively.

Previous studies have shown that transgenic plants harbor a few copies of T-DNA insertions, with an average of 1.2–2.0 copies, and most have only a single copy [27, 28]. In the present study, various homozygous lines of the T_2_ generation harbored 1–2 copies of T-DNA, and the insertion sites of T-DNAs varied. This also confirms that T-DNA insertion is a relatively random event [29, 30].

### Overexpression of exogenous *βAS* in transgenic rice

**Fig. 5:**
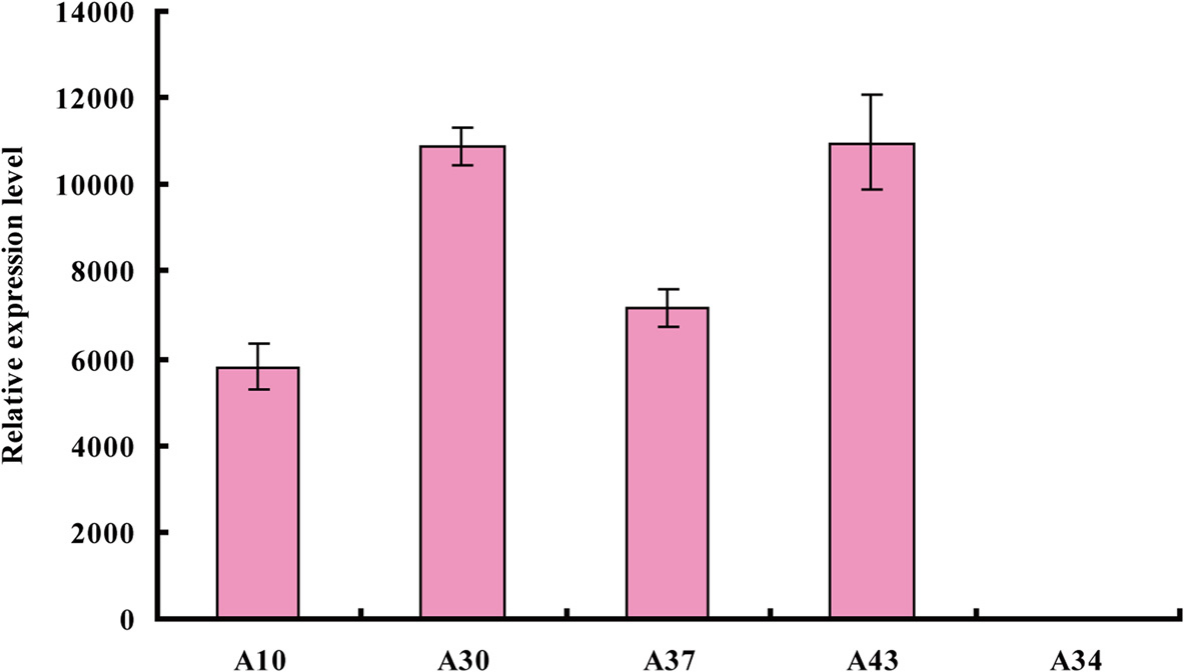
Real-time fluorescence quantitative PCR analysis of *βAS* expression in transgenic rice plants

Real-time PCR analysis showed that the *βAS* gene was overexpressed in the T_2_ generation homozygous lines A10, A30, A37, and A43. The relative expression level was within the range of 5782.1–10957.8 (Fig. 5, A34 was a negative line).

### Western blot analysis of *β*AS protein

**Fig. 6:**
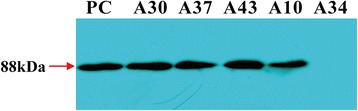
Detection of the *β*AS protein (about 88 kDa) in transgenic rice by western blotting. PC, positive control (the products of prokaryotic expression of *βAS*). A34, negative rice control

The rice positive samples of the T_2_ generation homozygous lines A10, A30, A37, and A43 and the positive control (prokaryotic expression product of *βAS*) showed the expected hybridization band of about 88 kDa in size. This specific band was not detected in the negative sample A34 line (Fig. 6). These results indicated that the seeds of the transgenic rice expressed *β*AS.

### Analysis of sapogenin content in transgenic rice by HPLC

**Fig. 7:**
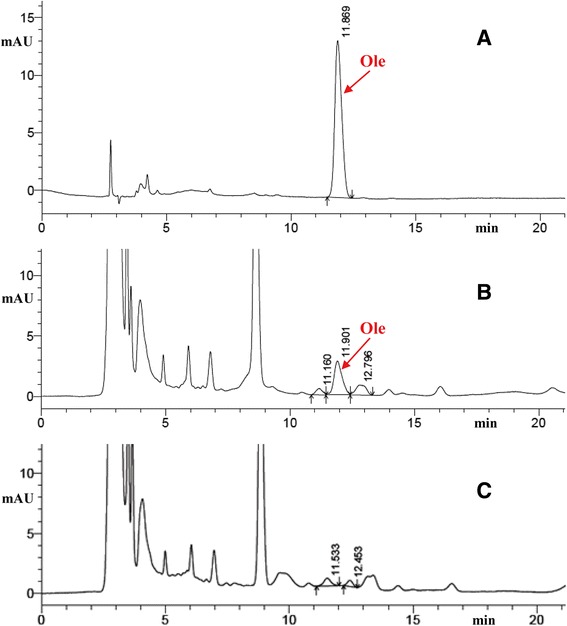
HPLC chromatograms of oleanolic acid. **a** Ole standard. **b** HPLC chromatograms of Ole in transgenic rice plants. **c** non-transgenic control rice

**Fig. 8:**
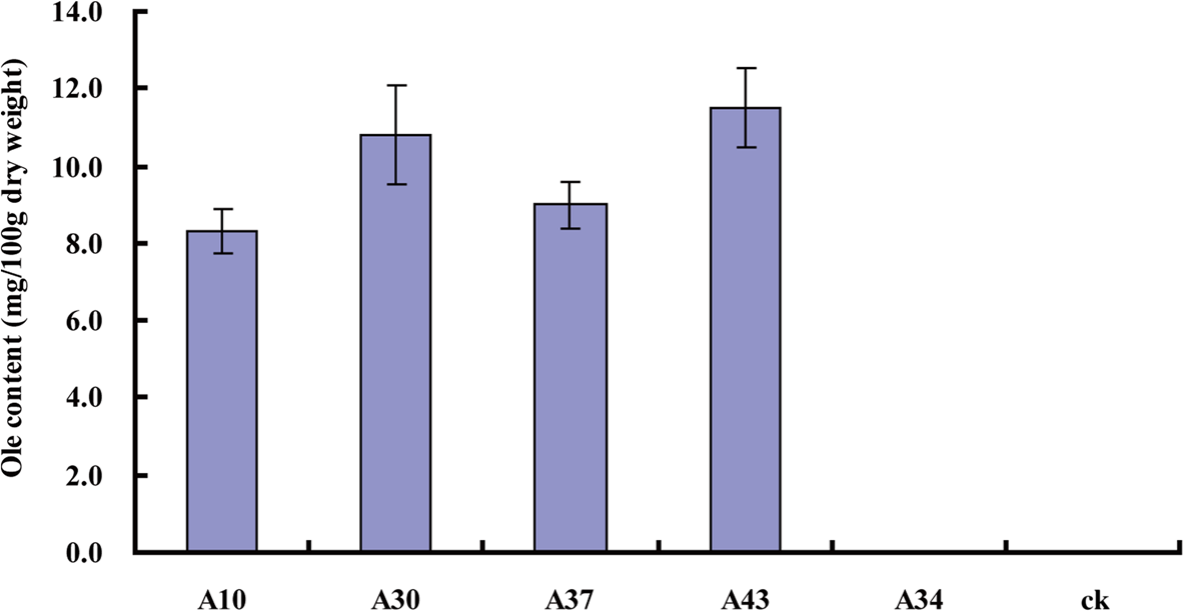
Oleanolic acid production as analyzed by HPLC. (ck), non-transgenic control rice

HPLC analysis indicated that the seeds of the T_2_ generation homozygous lines A10, A30, A37, and A43 had an Ole content ranging from 8.3 to 11.5 mg/100 g dw (Figs. 7 and 8).

### Transgenic yeast and rice as new sources of ginsenosides

Because supplies of wild ginseng have been depleted, ginsenosides are mainly extracted from cultivated ginseng. However, artificial cultivation is relatively time-consuming and does not allow continuous cropping, preventing ginsenoside production from meeting the increased demand of the community. Therefore, it is necessary to explore new sources of ginsenosides for medicinal use [8, 9]. Scientists utilize synthetic biology to synthesize ginsenosides. Cloning and characterization of a key enzyme in the synthesis of 20 (S)-protopanaxadiol (PPD) has resulted in the construction of a yeast cell capable of producing PPD [31]. Cytochrome P450 was found to catalyze PPD to produce 20 (S)–protopanaxatriol (PPT) [32]. A “ginseng yeast” capable of simultaneously producing three kinds of ginsenosides, Ole, PPD, and PPT has also been constructed, with yields of 21.4 mg/L, 17.2 mg/L, and 15.9 mg/L, respectively [33, 34]. The rare ginsenoside, compound K (CK), which has not been detected in ginsenosides, is the active metabolite that has been detected in the blood after oral administration of ginseng and other ginsenosides [35]. Yan *et al.* previously reported that ginseng UDP-glycosyltransferase (PgUGT1) could specifically catalyze glycosylation of the C-20S hydroxyl group in dammarane-type tetracyclic triterpenoids, and yeast cells synthesized CK from monosaccharides when PgUGT1 was co-expressed in the PPD synthesis pathway [36].

Adding the biosynthetic branch of new metabolites to rice by using genetic engineering facilitates the synthesis of new active ingredients. Modifying existing metabolic pathways in rice to increase the concentration of specific components to improve its nutritional value has become an important means of creating new germplasm. A number of important advances such as “golden rice” [5, 6], “high-iron rice” [37, 38], and “sesame nutrition rice” [7] can be attributed to this technique. In the present study, a new rice germplasm that produces oleanane-type sapogenin was produced by expressing the *P. japonicus βAS* gene. This germplasm can serve as a new resource for breeding “ginseng rice” varieties, as well as create new ginseng saponin donors. Because rice is a food crop that is simpler to cultivate than the ginseng genus plants, it is generally easier to produce saponins in modified strains, thus allowing more people to benefit from this novel production approach. Improvement in the standard of living has resulted higher production requirements for medicine and food. The saponins synthesized by plants might be safer and more efficient than those produced via cellular engineering. Therefore, the results of the present study will have a major impact on the genetic breeding of functional rice, as well as on research and development of sources of medical ginsenosides.

## Conclusions

This is the first report on the transformation of the *P. japonicus βAS* gene into rice and the generation of a new “ginseng rice” germplasm that produces oleanane-type sapogenin. The concentration of Ole in transgenic rice was within the range of 8.3–11.5 mg/100 g dw. The current results indicate that it is feasible to breed new varieties of “ginseng rice” and create new ginsenoside donors by transforming key genes in the ginsenoside biosynthesis pathway.

## Abbreviations

*β*AS: *β*-Amyrin synthase
T-DNA: Transfer-DNA
*P. japonicus*: *Panax japonicus*
*P. ginseng*: *Panax ginseng*
*P. quinquefolius*: *Panax quinquefolius*
FPP: Farnesyl pyrophosphate
SQS: Squalene synthase
SQE: Squalene epoxidase
CS: Cycloartenol synthase
LS: Lanosterol synthase
*α*AS: *α*-Amyrin synthase
LUS: Lupeol synthase
DS: Dammarenediol-II synthase
OSCs: 2,3-Oxidosqualene cyclases
35S polyA: Terminator of *CaMV* 35S gene
*HPT*: Hygromycin phosphotransferase gene
35S P: 35S promoter
NOS T: Terminator of the nopaline synthase gene
Ubi P: Ubiquitin promoter
LB: Left border
RB: Right border
*E. coli*: *Escherichia coli*
*A. tumefaciens*: *Agrobacterium tumefaciens*
Hyg: Hygromycin B
hiTAIL-PCR: High-efficiency thermal asymmetric interlaced PCR
HPLC: High-performance liquid chromatography
SDS-PAGE: Sodium dodecyl sulfate polyacrylamide gel electrophoresis
PC: Positive control
Ole: Oleanolic acid
PPD: 20 (S)-Protopanaxadiol
PPT: 20 (S)–Protopanaxatriol
CK: Compound K
PgUGT1: *Panax ginseng* UDP-glycosyltransferase
